# Dynamics of Sundarban estuarine ecosystem: eutrophication induced threat to mangroves

**DOI:** 10.1186/1746-1448-6-8

**Published:** 2010-08-11

**Authors:** Suman Manna, Kaberi Chaudhuri, Somenath Bhattacharyya, Maitree Bhattacharyya

**Affiliations:** 1 Institute of Environmental Studies and Wetland Management, DD-24, Sector-I, Salt lake, Kolkata-700064, India; 2 Department of Biochemistry, University of Calcutta, 35, B.C. Road, kolkata-700019, India

## Abstract

**Background:**

Sundarbans is the largest chunk of mangrove forest and only tiger mangrove land in the world. Compared to the rich species diversity and uniqueness, very few studies have so far been conducted here, mainly due to its inaccessibility. This study explores water quality, density of biomass, species diversity, phytoplankton abundance and bacterial population of a tidal creek in Sunderban estuary during the post and pre monsoon period of 2008-09.

**Results:**

Phytoplankton community was observed to be dominated by diatoms (Biacillariophyceae) followed by Pyrrophyceae (Dinoflagellates) and Chlorophyceae. A total of 46 taxa belonging to 6 groups were recorded. Other algal groups were Cyanophyceae, Euglenophyceae and Chrysophyceae. Species diversity was highest in summer (March) and lowest in winter season (November) in all the sample stations indicating its close correlation with ambient temperature. Species evenness was fairly high in all five stations throughout the study period. Present study indicated that dissolved oxygen, nutrients and turbidity are the limiting factors for the phytoplankton biomass. The estuary was in eutrophic condition (Chlorophyll-a ≥10 μg/L) in winter. During the month of May phytoplankton biomass declined and at high salinity level (21.2PSU) new phytoplankton species take over, which are definitely better resilient to the high saline environment. Bio-indicator species like *Polykrikos schwartzil, Dinophysis norvegica and Prorocentrum concavum *points to moderately polluted water quality of the estuary.

**Conclusion:**

Eutrophication as well as presence of toxic Dinoflagellates and Cyanophyceae in the tidal creek of Sundarban estuary definitely revealed the deteriorated status of the water quality. The structure and function of the mangrove food web is unique, driven by both marine and terrestrial components. But little attention has been paid so far to the adaptive responses of mangrove biota to the various disturbances, and now our work unfolds the fact that marine status of Sundarban estuary is highly threatened which in turn will affect the ecology of the mangrove. This study indicates that ecosystem dynamics of the world heritage site Sundarban may facilitate bioinvasion putting a question mark on the sustainability of mangroves.

## Background

Sundarban is the single largest chunk of mangrove forest in the world. Formed at estuarine phase of the Ganges - Brahmaputra river system, the Sundarban ecosystem is unique in many respects. The area experiences a subtropical monsoon climate with the annual rainfall of about 1600-1800 mm and several cyclonic storms. This mangrove ecosystem of Indian subcontinent is well known not only for the aerial extent, but also for the species diversity. The biodiversity of Sundarban includes numerous species of phytoplankton, zooplankton, micro-organisms, benthic invertebrates, mollusks, amphibians and mammals [1]. It is the only mangrove tiger land on the earth. It has been declared as a world heritage site by International Union for Conservation of Nature (1987). However, the landscape of the Sundarbans have changed remarkably due to neo-tectonic movement compounded with large scale human intervention from the beginning of last centaury, as a result several species have become extinct or are in very much threatened or degraded state [1,2]. But any systematic approach towards studying the ecosystem dynamics of Sundarban has not been attempted so far [1,3].

Our work attempts to explore and understand the correlation between different components of Sundarban ecosystem. The study area is located in the reserved mangrove forest of Sundarbans within the 24-Parganas Forest Division. Herobhanga Forest Block, the northern-most block out of seven forest blocks covers over an area of about 200 km^2^. Out of this, about 16 square kilometers of mangroves were lost due to encroachment and human intervention over a period of about last fifteen years. Total mangrove coverage in this block was 73.05 km^2 ^as per Survey of India topographic sheet (Surveyed in 1988-89) which was dwindled to 57.53 km^2 ^as deciphered through land use land cover study using remote sensing data of the year 2006 (IRS P-6, LISS-IV data). The entire loss of mangrove coverage was recorded on the northern side of the Bara Herobhanga Khal adjacent to the inhabited Jharkhali Island. In fact, out of nine Forest compartments present in Herobhanga Forest Block, compartment numbers 1 (one), 2 (two) and 3(three) have been completely reclaimed and converted into either aquaculture ponds or agricultural land. Effluents from these aquaculture ponds are disposed through another small creek into this Bara Hero Bhanga Khal (creek), thus acting as a point source contaminant into this mangrove ecosystem; while rain washings from the agriculture fields mix up with this Bara Hero Bhanga Khal as a non-point source of impure water. At the same time, this particular creek separates inhabited Jharkhali Island from the Herobhanga Forest, which is a dense mangrove forest having hardly any human intervention.

This creek also joins two mighty tidal rivers of Sundarbans, namely Matla River in the west and Bidya River in the east and plays an important agent for hydrodynamic set up of this area. Thus, this creek along which sampling and analysis of water was carried out in pre and post-monsoon time for one-year period represents both pristine environment along its south bank (the border with dense mangrove forest) and human interfered environment along its north bank (the border with inhabited Jharkhali Island). During different seasons of the year water quality was studied and the concentrations of the nutrients like ammonia, nitrite, silicate and phosphate was quantitated. Primary producers in this estuarine ecosystem was characterized and the dominant species was identified. Phytoplankton is good indicator of trophic states and many species of this community are sensitive to environmental changes. Their presence or absence from the community indicates changes in physio-chemical environment of the estuary [4]. Seasonal distribution patterns of phytoplanktons and primary producers were investigated thoroughly accompanied with the bacterial abundance in the estuarine water. It was envisaged that analysis of different physico-chemical as well as microbial parameters of water samples along different points on both sides of this river might throw some light on the effect of human intervention on the Sundarban eco-system to make the study significant.

## Methods

### Study area

**Figure 1:**
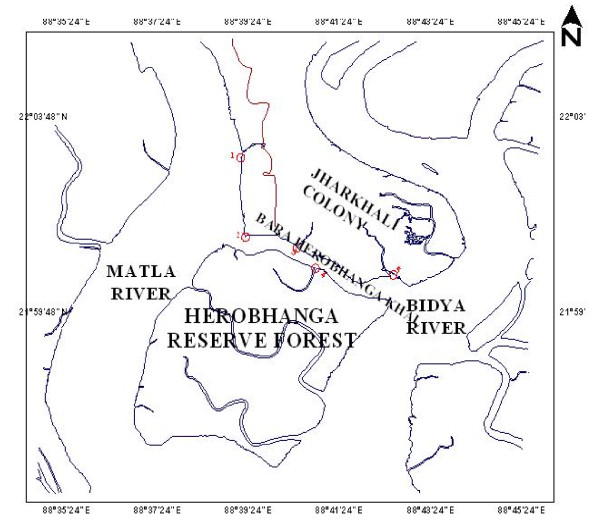
**Map of the Jharkhali tidal creek with marked sampling points**.

The exact area for this study was in Jharkhali island, a small locality about 130 km from Kolkata (Figure 1). The area is located in Survey of India topographic sheet no. 79 B/12. A small creek (about 150 meter wide) known as 'Bara Herobhanga Khal' separates the Jharkhali Island from Herobhanga Reserve Forest (which is within Project Tiger area). The 'Bara Herobhanga Forest' joins two mighty river of Sundarbans namely the Matla River on the West and the Bidya River in the East. Matla is connected to Bidya and ultimately flows to the Bay of Bengal. The fresh water connection and discharge to this river has been lost in recent times. Salinity of the river water is relatively high owing to fresh water cutoff from upstream region. There is no previous physico-chemical or biological investigation of this tidal creek (River Matla, Herobhangakhal and adjoining places).

### Description of study sites

Baro Herobhanga Khal is a creek which joins Matla River in the west and the Bidya River in the east. Both these rivers have no upland freshwater discharge now and have been transformed into mere tidal creeks. All these five sample points are distributed along the junctions of these rivers with the Bara Herobhanga Khal and along the stretch of the Bara Herobhanga Khal itself.

Sample Point No. 1: It is on the Matla River itself. A small creek from the Forest Jetty joins Matla at this point.

Sample Point No. 2: It is just at the junction of the Matla River with Bara Herobhanga Khal near the margin of the river. It is about 3.180 km north of 1st Sample point along the river.

Sample Point No. 3: It is near the concrete jetty at Jharkhali village within the Bara Herobhanga River near the northern margin. It is about 2.170 km towards east of sample point no. 2.

Sample Point No. 4: It is located within the small creek going inside mangrove forest on the southern side of the Bara Herobhanga River. It is about 1.2 km south-east of point no.3.

Sample Point no. 5: It is near the junction point of Bara Herobhanga Khal with the Bidya River, another mighty tidal river just like Matla River, running approximately in the North-South Direction.

This Bara Herobhanga River is extremely significant since both Matla and Bidya Rivers are mighty rivers running along Nort-South direction in Sundarban. The tide comes simultaneously through both the rivers. This Bara Herobhanga River joins both the rivers in an East-West direction. It is of interest to investigate which river system dominates over the other through such connection. This study may throw some light on this direction. However, it needs further investigation, especially hydrodynamic studies which may indicate what happens in such cases. It is to be noted here that Sundarban is a network of rivers, where such comparatively smaller creeks join the larger rivers forming a deltaic environment.

### Sample collection

Five stations were set up in the tidal creek to capture the overall diversity of the estuarine ecosystem in and around Jharkhali island. The details of the five stations are given in Table 1.

**Table 1:** Sample points.

Details of sample points	Latitude	Longitude
Station 1	22°02' 49.1758"	88°39' 39.8203"

Station 2	22°01'25.6022"	88°39' 52.0801"

Station 3	22°01'07.7800"	88°40' 55.8051"

Station 4	22°00'41.2819"	88°41' 17.4375"

Station 5	22°00'37.5904"	88°42' 55.0400"

Specific details of the sample points with latitude and longitude.

Field trips were conducted at fortnight intervals to collect samples at the points that were either measured on board of the launch or were brought back to laboratory for analysis. This study period extended from November to February (post monsoon study) and March to May (pre monsoon study). Samples were collected from the water surface (0.5 m depth) of all the five stations along the Matla and Bidya river. The water was filtered through 25 μm Nitex mesh at 10-15 mm of Hg vacuum to remove larger organisms and debris. Samples were also collected on deeper cast and filtered appropriately (Membrane filters 0.45 μm) using a Millipore suction apparatus for the study of water quality parameters. All samples were preserved in cold condition, transported to the laboratory within three hours of collection to analyze immediately.

### Physico-chemical analysis

Water temperature, pH and conductivity were measured *in situ *with Hach Portable Meters (HQ40d). Turbidity was measured by using portable turbidity meter (Hach 2100P), salinity was determined in practical salinity units by Knudsen method [5], dissolved oxygen concentration was studied according to the method of Winkler [6], nutrients like inorganic nitrogen (ammonia, nitrite), soluble phosphate and reactive silicate were measured according to the same methodology [6].

### Biological analysis

#### Phytoplankton biomass (Chlorophyll-a)

Chlorophyll samples were drawn from all stations, with a maximum vertical spacing of 10 m through the chlorophyll maximum layer, at least one sample was always taken within 5 m of the maximum concentration. Chlorophyll samples were filtered through Whatman GF/F (0.45 μ) filters and extracted in acetone in dark and refrigerated condition. Chlorophyll-*a *was determined spectrofluorimetrically [7].

#### Total count of Phytoplankton and Bacteria

Fluorescence microscope was used to estimate the total number of phytoplankton and bacteria. Immediately after sampling, 50 ml of seawater was preserved with 25% gluteraldehyde (0.2-μm-prefiltered) and stored in cold dark environment to prevent reduction of counts. Cells of phytoplankton and bacteria were collected onto a 25-mm black polycarbonate Nucleopore membrane with a 0.45 μm and 0.2 μm pore size respectively and stained with Acridine orange. Slides were preserved at -20°C until they were counted. Twenty random fields were counted in a Zeiss confocal fluorescence microscope coupled with an image analysis system [8,9]. Direct estimation of phytoplankton cell count was also performed using Sedgwick-Rafter counting chamber [10]. Viable count of Bacterial colonies was also performed using Luria-Bertani medium by serial dilution method [11].

### Community structure analysis

Direct estimation of phytoplankton cell abundance and diversity was performed by cell counting method. Surface phytoplankton was collected and the Lugol's preserved subsamples (1-2 liter) were used for quantitative enumeration utilizing a Sedgwick-Rafter counting chamber and Zeiss research microscope according to UNESCO PROTOCOL [10]. Three indices were used to obtain the estimate of the species diversity (H^1^), species richness (d) and species evenness (J).

Shannon & Wiener [12] diversity index value was obtained using the following equation:

$${\text{H}}^{1}=\sum_{\text{I}=1}{\text{p}}_{\text{i}}\log_{\text{e}}{\text{p}}_{\text{i}}$$

Where H^1 ^= Shannon & Wiener diversity index

P_i _= Proportion of sample made up by the ith species

S = Total number of species

Species richness (d) was obtained using the equation

d=S−1/lnN

Where d = Margalef's diversity index [13]

S = Total number of species

N = No of individuals

Species evenness was determined by using the expression of Pielou [14]

$$\text{J}={\text{H}}^{1}/\text{ln}\text{S}$$

Where H^1 ^= Shannon and Wiener index

J = Evenness

S = Total number of species

### Identification of phytoplankton

Surface water samples were collected using plankton net (20 μm) and immediately fixed with Lugol,s solution and buffered formaldehyde. The preserved samples were kept in refrigerator until analysis. Before identification water samples were allowed to settle for 24 hours and the supernatant was decanted until a concentrate 10 ml was achieved. Few drops of concentrated sample were taken in a common glass slide and observed under Zeiss binocular microscope equipped with phase contrast optics and photographed with Cannon A 1000 camera. In most of the time phase contrast optics was used because it revealed especially well lightly silicified cells of diatoms. For identification of common diatoms examination of raw (without acid cleaned) material in a water mount was done as stated earlier. But for identification of diatoms with specialized structure like striation (*Navicula sp*) or raphe (*Pseudonitzchia sp*) acid clearing of samples was adopted which was used to separate diatoms frustules into single valves on which structure diatoms were best seen. Several keys and illustration were consulted to confirm identification [15-22].

### Statistical analysis

The results were expressed as differences between the groups considered significant at p < 0.05. Data comparison and influence of the environmental factors on phytoplankton were evaluated by stepwise multiple regression [23] Different statistical analysis and correlation regression analysis were performed using the software STATISTICA.

## Results

### Physical and Chemical analysis

**Figure 2:**
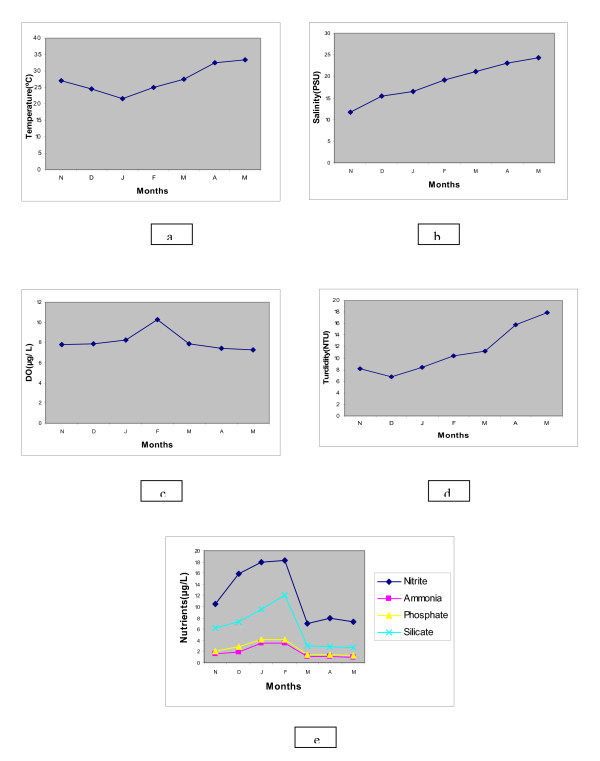
**Seasonal variation in (a) temperature (°C), (b) salinity (PSU), (c) dissolved oxygen (mg L^-1^), (d) turbidity (NTU) and (e) nutrients (μg L^-1^) of estuary**. Points represent mean of twenty samples in every month.

Temperature of the surface water varied continuously through post monsoon to pre monsoon (Nov'08-May'09) (Figure 2a). The lowest surface water temperature 21°C was recorded in January (2009) while the highest 33°C was recorded in May (2009). pH of the water sample was weakly alkaline and more or less constant in the range of 8.0-8.15 throughout the study period. Salinity level gradually increased from post monsoon to pre monsoon period in the range 10.6-24.6 PSU, lowest value being recorded in November and the highest in May. A steady increase in salinity from station 1 to station 5 was observed in all the sampling months. Average salinity of winter months (Nov-Feb) was 16.17 PSU, lower compared to summer months (March'09-May'89) (23.5PSU) (Figure 2b).

Moderate to high dissolved oxygen concentration was observed throughout the sampling months with its maximum in February. Dissolved oxygen concentration was observed to be 8.5 mg L^-1 ^in average in winter and 7.5 mg L^-1 ^in summer (Figure 2c).

Turbidity (NTU) is used to describe water clarity and the values were observed in the range of 6.05 NTU to 21.05 NTU (Figure 2d) during post monsoon to pre monsoon.

Nutrient level i.e. Nitrite-Nitrogen, ammonia-Nitrogen, phosphate and silicate showed higher concentration in winter months compared to summer months. Concentration of Phosphate and Ammonia-Nitrogen were always lower than Silicate and Nitrite. Nitrite-Nitrogen concentration of samples ranged from 7.05 μg/L in summer to 18.25 μg L^-1 ^in winter. Ammonia-Nitrogen concentration was estimated to be 6.17 μg L^-1 ^(average) in winter and 0.94 μg L^-1 ^(average) in summer. Phosphate concentration of water sample ranged from 3.44 μg L^-1 ^(in winter) to 1.41 μg L^-1 ^(in summer). Silicate concentration in surface water sample ranged from 8.17 μg L^-1 ^to 2.86 μg L^-1 ^in winter and summer respectively (Figure 2e).

### Biological Analysis

#### Biomass

**Figure 3:**
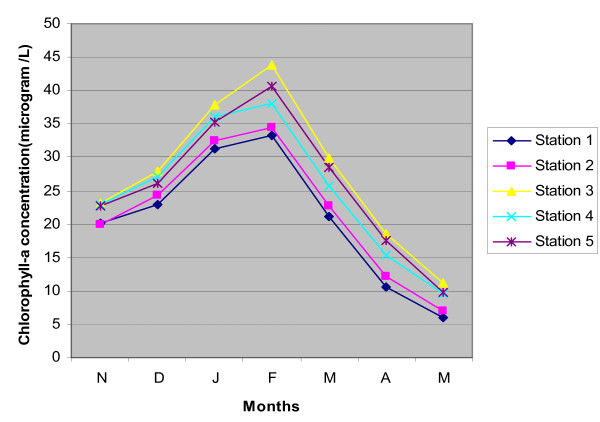
**Seasonal variation of chlorophyll-a concentration (μg L^-1^) of five sampling points throughout the study period (Nov'08-May'09)**.

**Figure 4:**
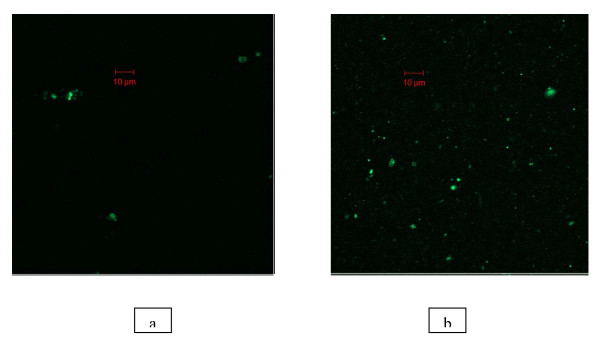
**Acridine Orange stained cells in microscope fields on black Nucleopore filters (a) Phytoplankton cells and (b) Bacterial cells**.

Chlorophyll-a concentration is an index of phytoplankton biomass and the concentration was estimated to be 5.9 μg L^-1 ^to 43.80 μg L^-1 ^through premonsoon to post monsoon months (Nov'08-May'09). Chlorophyll-a concentration was observed to be 33.15 μg L^-1 ^(average) in summer and 19.86 μg L^-1 ^(average) in winter. Chlorophyll-a concentration reached its maximum in February'09 in all the five sampling stations (Figure 3) and similar trend was observed for phytoplankton cell count. Phytoplankton cell count ranged from 1.80 × 10^4 ^cells L^-1 ^to 2.05 × 10^7 ^cells L^-1 ^with an average of 2.52 × 10^6 ^cells L^-1 ^in winter and 9.93 × 10^5 ^cells L^-1 ^in summer. Fluorescence count of phytoplankton cell ranged from 2.17 × 10^6 ^to 1.42 × 10^8 ^in winter and 2.2 × 10^5 ^to 4.9 × 10^6 ^in summer (Figure 4a, Table 2). The conventional counts for viable bacteria obtained on Luria-Bertani agar medium ranged from 3.68 × 10^6 ^CFU L^-1 ^to 2.64 × 10^7 ^CFU L^-1 ^during winter season and between 4.48 × 10^7 ^CFU L^-1^and 8.9 × 10^8 ^CFU L^-1 ^during summer. Direct counts of bacterial cells ranged from 8.54 × 10^7 ^cells L^-1 ^to 9.5 × 10^8 ^cells L^-1^cells in winter and between 1.0 × 10^9 ^cells L^-1^and 4.52 × 10^10 ^cells L^-1 ^in summer (Figure 4b, Table 3).

**Table 2:** Abundance of phytoplankton in Sundarban estuary.

Month	Phytoplankton Count					
	
	Lugol's Count (Cells L^-1^)			Fluorescence count (Cells L^-1^)		
	**Minimum**	**Maximum**	**Mean**	**Minimum**	**Maximum**	**Mean**

Nov'08	6.5 × 10^5^	1.2 × 10^6^	8.50 × 10^5^	1.5 × 10^6^	3.5 × 10^6^	2.17 × 10^6^

Dec'08	8.0 × 10^5^	1.1 × 10^6^	9.05 × 10^5^	1.0 × 10^6^	5.0 × 10^6^	2.6 × 10^6^

Jan'09	1.0 × 10^6^	5.5 × 10^6^	3.0 × 10^6^	1.72 × 10^7^	2.0 × 10^7^	1.57 × 10^7^

Feb'09	1.0 × 10^7^	3.96 × 10^7^	2.05 × 10^7^	5.0 × 10^7^	2.5 × 10^8^	1.42 × 10^8^

March'09	1.5 × 10^6^	2.5 × 10^6^	1.75 × 10^6^	3.0 × 10^6^	9.0 × 10^6^	4.9 × 10^6^

April'09	1.2 × 10^6^	2.0 × 10^6^	1.22 × 10^6^	1.8 × 10^6^	3.0 × 10^6^	1.9 × 10^6^

May'09	5 × 10^3^	3.5 × 10^4^	1.80 × 10^4^	1.4 × 10^5^	4.0 × 10^5^	2.2 × 10^5^

Abundance of phytoplankton in Sundarban estuary. Values represent mean of twenty samples in every month.

**Table 3:** Abundance of bacteria in Sundarban estuary.

Month	Bacterial count					
	
	Plate count **(CFU L**^**-1**^**)**			Fluorescence count **(Cells L**^**-1**^**)**		
	**Minimum**	**Maximum**	**Mean**	**Minimum**	**Maximum**	**Mean**

Nov'08	8.0 × 10^6^	5.2 × 10^7^	2.64 × 10^7^	5.0 × 10^8^	2.0 × 10^9^	9.50 × 10^8^

Dec'08	1.0 × 10^6^	1.0 × 10^7^	5.0 × 10^6^	7.0 × 10^7^	3.3 × 10^8^	1.62 × 10^8^

Jan'09	1.25 × 10^6^	6.75 × 10^6^	3.68 × 10^6^	6.0 × 10^7^	1.2 × 10^8^	8.54 × 10^7^

Feb'09	6.0 × 10^6^	3.4 × 10^7^	1.54 × 10^7^	3.0 × 10^8^	1.3 × 10^9^	7.60 × 10^8^

March'09	1.5 × 10^7^	8.5 × 10^7^	4.48 × 10^7^	5.0 × 10^8^	2.5 × 10^9^	1.0 × 10^9^

April'09	7.0 × 10^8^	1.0 × 10^9^	7.9 × 10^8^	2.0 × 10^10^	7.0 × 10^10^	4.14 × 10^10^

May'09	5.0 × 10^8^	1.5 × 10^9^	8.9 × 10^8^	2.0 × 10^10^	8.0 × 10^10^	4.52 × 10^10^

Abundance of bacteria in Sundarban estuary. Values represent mean of twenty samples in every month.

#### Species composition

**Figure 5:**
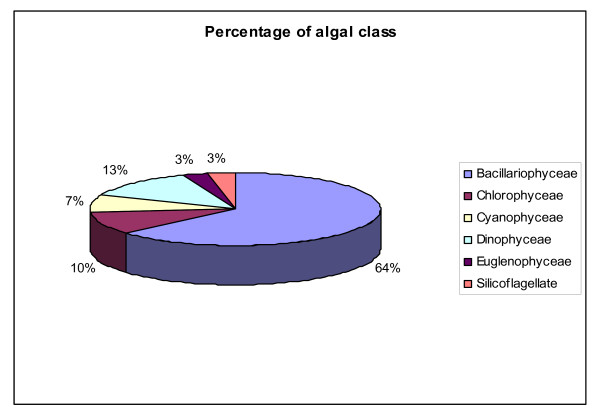
**Percentage of algal division in Sundarban estuary in the total study period (Nov'08-May'09)**.

**Figure 6:**
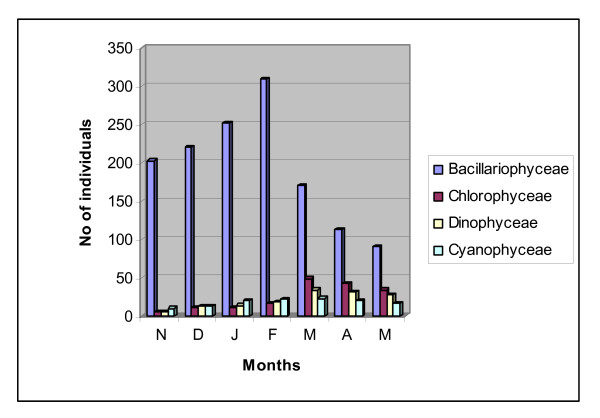
**Relative abundance (cells L^-1^) of planktonic classes over the study period (Nov'08-May'09)**.

**Figure 7:**
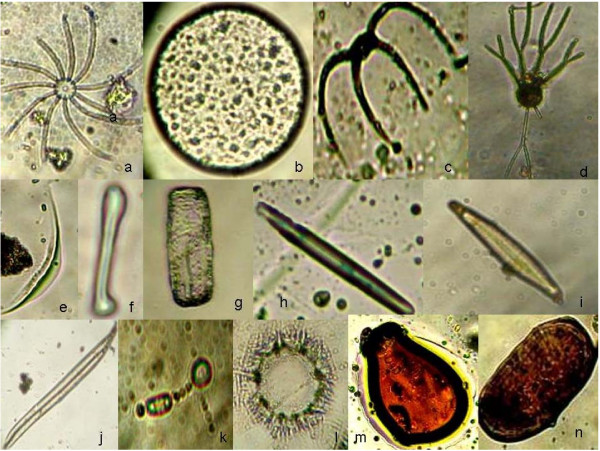
**Representative phytoplankton taxa identified in Sundarban estuary**. a) *Bacteriastrum sp *b) *Coscinodiscus sp *c) *Chaetoceros curvesetus *d) *Bacteriastrum hyalinum *e) *Closterium *f) *Asteronella *g) *Grammatophora marina *h) *Nitzschia sp *i) *Navicula penata *j) *Gyrosigma baticum *k) *Anabaena *l) *Asterionellopsis gracilis *m) *Dinophysis norvegica *n) *Polykrikos schwartzil*.

Six major classes were recorded in this study. Bacillariophyceae (Diatoms), Chlorophyceae (Green algae), Cyanophyceae (Blue green algae), Pyrrophyceae (Dinoflagellates) and Chrysophyceae (Figures 5 &6). The phytoplankton community was dominated by Diatoms. Out of 46 phytoplankton taxa identified, 27 genera (46 species) were Diatoms. Centrales were represented by 13 taxa containing 27 species and Pennales by 14 taxa containing 19 speceis. Centric Diatoms predominated in winter months while Pennates in summer (Figure 7). Most abundant pennate Diatoms were *Pinnularia, Navicula, Gyrosigma, Thalassionema and Climacosphenia *and most abundant centric Diatoms were *Chaetoceros, Coscinodiscus, Cyclotella, Bacteriastrum, Actinocyclus and Planktoniella*. Pyrrophyceae (Dinoflagellates) made up of 6 taxa containing 9 speceis. Among Pyrrophyceae ((Dinoflagellates) 6 genera were *Prorocentrum, Protoperidinium, Peridinium, Ceratium, Dinophysis and Polykriskos. Protoperidinium, Dinophysis and Ceratium *were the most abundant genera in Pyrrophyceae (Table 4). The abundance of Dinoflagellates (Table 5) were slightly higher in pre monsoon (99 × 10^3^) than post monsoon (59 × 10^3^). The green algal forms (Chlorophyceae) comprised of 6 genera namely *Cosmarium, Closterium, Netrium, Chorella, Dunaliella, and Drapernaldia*. Blue-green algae (cyanophyceae) comprised of 5 genera namely *Anabaena, Stigonema, Oscillatoria, Gleocapsa and Trichodesmium*. Euglenoids were represented by only one taxa *Euglena and Crysophyceae by two species of single genus Dictyocha*. (Table 4). In the sampling stations 1 and 2 species composition was similar. Interestingly, in stations 3 and 4 species composition were also similar. This might be due to proximity of stations 1 & 2 and 3 & 4. The stations 3 and 4 received nutrient rich discharges from aquaculture ponds. This area was also subjected to anthropogenic influence due to tourism. As a result the pollution level in water in station 3 and 4 were higher. This was reflected in the presence of higher frequency of Dinoflagellates there. The station 5 showed different species compositions with higher abundance of salinity tolerance species, Chlorophyceae and Cyanophyceae. Station 5 was located towards the sea and had higher salinity level, which influenced the species composition here (Table 6). In general higher planktonic biomass was recorded in winter months than summer. Occurrence of *Prorocentrum concavum *from the estuary deserves special mention here because its occurrence was reported in sub-tropical mangrove habitats [24] outside India so far our knowledge concern. This is the first reporting when *Prorocentrum concavum *has been identified in Sundarban, the main mangrove in India.

**Table 4:** Phytoplankton composition

Identity of taxa	Average no of individuals (cells/ml)						
	
	Nov, 08	Dec, 08	Jan, 09	Feb, 09	March, 09	April, 09	May, 09
**Class **Bacillariophyceae							
**Order**							
**Centrales**							
*Coscinodiscus radiatus*	18	17	18	22	8	7	7
*Coscinodiscus perforatus*	14	13	15	19	2	1	1
*CoscinodiscusIII*	13	14	22	18	1	-	-
*Cyclotella sp.*	5	5	4	16	-	-	-
*Cyclotella striata*	13	14	16	18	2	-	-
*Triceratinum spI*	15	18	20	24	6	2	1
*Triceratinum spII*	3	4	8	12	2	1	-
*Paralia sulcata*	2	3	8	10	1	-	-
*Asteromphalus sp*	2	2	4	6	3	1	-
*Hyalodiscus sp*	2	2	2	4	2	-	-
*Thalassiosira punctigera*	14	16	16	19	4	2	2
							
*Thalassiosira spII*	2	3	3	6	1	-	-
*Bascteriastrum hyalinum*	16	17	16	18	4	2	2
*Bascteriastrum spII*	4	5	5	8	2	-	-
*Actinocyclus octanarius*	8	8	10	18	2	1	-
*Actinocyclus spII*	6	9	11	14	2	1	1
*Actinoptychus sp*	3	4	4	7	1	-	-
*Chaetoceros curvisetus*	16	16	18	18	3	2	2
*Chaetoceros subtilis*	15	16	18	16	5	1	3
*Chaetoceros convolutus*	15	14	13	20	3	3	3
*ChaetocerosIV*	13	5	6	13	4	2	1
*ChaetocerosV*	4	6	6	16	3	1	1
*ChaetocerosVI*	4	4	4	8	4	1	1
*ChaetocerosVII*	3	4	18	6	2	-	-
*Planktoniella spI*	8	10	18	14	1	-	-
*Planktoniella spII*	6	12	10	18	1	1	-
*Eunotia sp.*	5	7	9	12	7	3	1
**Order**							
**Pennales**							
*Navicula penata*	2	2	1	2	12	6	6
*Navicula spII*	-	2	1	2	10	8	6
*Pinnularia spI*	-	-	-	2	9	5	5
*Pinnularia spII*	-	1	1	2	12	6	5
*Diatoma sp*	-	-	-	6	16	8	4
*Tabellaria sp*	3	2	2	6	10	9	2
*Fragillaria sp I*	-	-	-	4	4	4	3
*Fragillaria sp II*	-	1	1	7	15	6	3
*Gyrosigma baticum*	-	1	1	2	13	4	3
*Grammatophora marina*	1	1	1	2	12	5	2
*Climatopleura sp*	-	-	-	1	5	4	3
*Climacosphenia elongata*	1	-	-	2	6	5	3
*Climacosphenia spII*	-	1	2	4	16	8	2
*Cymbella marina*	1	1	1	2	4	10	3
*Thalossionema nitzschioides*	4	5	6	11	19	8	5
*Thalossionema sp*	2	3	3	8	8	8	3
*Nitzschia spI*	1	1	-	2	18	8	3
*Asterionella sp*	1	4	2	4	5	5	2
*Asterionellopsis gracilis*	-	1	1	2	3	2	2
**Class Chlorophyceae**							
**Order**							
**Zygnimataes**							
*Cosmarium sp*							
*Closterium sp*	6	8	10	15	8	6	3
*Netrium sp*	1	2	2	4	6	4	2
**Order**	2	3	6	4	2	2	3
**Chlorococcales**							
*Chlorella salina*							
*Chlorella marina*	-	-	-	2	8	10	9
**Order**	1	2	2	3	11	6	6
**Dunaliellales**							
*Dunaliella salina*							
**Order**	-	-	-	1	14	17	19
**Chaetophorales**							
*Draparnaldia sp*							
**Class Pyrrophyceae**	-	1	2	4	6	6	3
**Order**							
**Prorocentrales**							
*Prorocentrum concavum.*							
							
**Order**	1	1	2	4	6	4	2
**Peridiniales**							
*Protoperinidium pellucidum*							
*Protoperinidium conicum*							
*Protoperinidium spIII*	2	2	1	3	5	6	4
*Peridinium sp*	1	1	1	1	4	6	3
*Ceratinum fusus*	1	1	1	1	2	4	2
	2	2	2	2	2	4	1
**Order**	1	1	1	1	1	3	2
**Dinophysales**							
*Dinophysis acuta*	-	2	2	4	6	2	4
*Dinophysis norvegica*	-	-	-	1	5	3	5
*polykriskos schwartzil*	2	3	4	5	5	4	4
							
**Class Cyanophyceae**							
**Order**							
**Nostocales**							
*Anabaena*	2	3	2	4	4	4	4
**Order**							
**Chrococcales**							
*Gleocapsa sp*	1	2	6	3	4	5	2
**Order**							
**Stigonematalis**							
*Stigonema sp*	3	2	2	4	5	5	5
**Order**							
**Oscillatoriales**							
*Oscillatoria sp*	3	2	4	5	5	7	8
*Tricodesmium sp*	1	4	6	6	6	2	2
							
**Class Euglenophyceae**							
**Order**							
**Euglenales**							
*Euglena spI*	4	6	4	7	7	5	2
*Euglena spII*	2	6	8	7	8	3	2
**Class Chrysophyceae**							
**Order**							
**Dictyochales**							
*Dictyocha speculum*	2	1	4	6	4	2	1
*Dictyocha spII*	2	4	6	6	2	1	1

Phytoplankton composition of most abundant taxa throughout the entire study period.

**Table 5:** Abundance of Dinoflagellates

Species	**Abundance (cells L**^**-1**^**)**	
	
	Post monsoon	Premonsoon
*Prorocentrum concavum*	8 × 10^3^	12 × 10^3^

*Protoperinidium pellucidum*	8 × 10^3^	15 × 10^3^

*Protoperinidium conicum*	4 × 10^3^	13 × 10^3^

*Protoperinidium spIII*	4 × 10^3^	8 × 10^3^

*Peridinium sp*	8 × 10^3^	7 × 10^3^

*Ceratinum fusus*	4 × 10^3^	6 × 10^3^

*Dinophysis acuta*	8 × 10^3^	12 × 10^3^

*Dinophysis noevegica*	1 × 10^3^	13 × 10^3^

*polykriskos schwartzil*	14 × 10^3^	13 × 10^3^

Abundance of Dinoflagellates in the estuary throughout the study period.

**Table 6:** Abundance and composition of phytoplankton taxa.

No. of station	Abundant taxa
Station 1	*Coscinodiscus spp., Cyclotella spp., Triceratinum spp., Hyalodiscus sp., Thalassiosira sp., Asteromphalus sp., Bascteriastrum sp., Actinocyclus spp., Actinoptychus sp., Chaetoceros spp., Planktoniella spp., Navicula spp., Pinnularia spp., Diatoma sp, Fragillaria spp., Gyrosigma sp., Climacosphenia spp., Nitzschia sp., Climatopleura sp., Thalossionema spp., Asterionellopsis sp., Cosmarium sp., Netrium sp., Ceratinum sp., Anabaena sp., Euglena spp., Dictyocha spp*.

Station 2	*Coscinodiscus spp., Cyclotella spp., Triceratinum spp., Paralia sp., Thalassiosira sp., Asteromphalus sp., Bascteriastrum sp., Actinocyclus spp., Actinoptychus sp., Chaetoceros spp., Planktoniella spp., Navicula spp., Eunotia sp., Diatoma sp., Tabellaria sp., Gyrosigma sp., Nitzschia sp., Climatopleura sp., Thalossionema spp., Asterionella sp., Closterium sp., Netrium sp., Protoperinidium spp., Oscillatoria sp., Dictyocha spp*.

Station 3	*Coscinodiscus spp., Cyclotella spp., Triceratinum spp., Thalassiosira sp., Bascteriastrum sp., Actinocyclus spp., Chaetoceros spp., Planktoniella spp., Navicula spp., Pinnularia spp., Fragillaria spp., Gyrosigma sp., Climacosphenia spp., Thalossionema spp., Asterionellopsis sp., Asterionella sp., Nitzschia sp., Eunotia sp., Draparnaldia sp., Dunaliella sp., Ceratinum sp., Prorocentrum sp., Protoperinidium spp., Peridinium sp., Dinophysis sp., polykriskos sp., Gleocapsa sp., Tricodesmium sp., Euglena spp., Dictyocha spp*.

Station 4	*Coscinodiscus spp., Cyclotella spp., Triceratinum spp., Thalassiosira sp., Bascteriastrum sp., Actinocyclus spp., Chaetoceros spp., Planktoniella spp., Navicula spp., Pinnularia spp., Fragillaria spp., Tabellaria sp., Gyrosigma sp., Climacosphenia spp., Thalossionema spp., Asterionellopsis sp., Asterionella sp., Nitzschia sp., Closterium sp., Dunaliella sp., Ceratinum sp., Prorocentrum sp., Protoperinidium spp., Peridinium sp., Dinophysis sp., polykriskos sp., Stigonema sp., Tricodesmium sp., Euglena spp*.

Station 5	*Coscinodiscus spp., Triceratinum spp., Bascteriastrum sp., Chaetoceros spp., Navicula spp., Diatoma sp., Nitzschia sp., Thalossionema spp., Cymbella marina, Grammatophora marina, Dunaliella salina, Chlorella salina, Chlorella marina, Draparnaldia sp, Cosmarium sp., Closterium sp., Netrium sp., Dinophysis sp., Anabaena sp., Gleocapsa sp., Stigonema sp., Tricodesmium sp., Oscillatoria sp*.

Abundance and composition of phytoplankton taxa in five sampling stations throughout the study period.

#### Community structure

**Figure 8:**
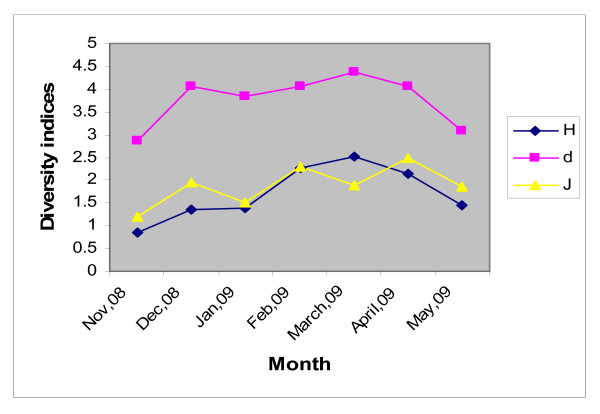
**Seasonal variation of diversity index (H), evenness (J) and richness (d) of the estuary**. Points represent mean of twenty samples in every month.

Phytoplankton cell count (cell density) was higher in winter months than summer. Lowest count was observed in May, 2009 and highest in Feb, 2009 in all the five sampling locations. Species richness (d) and species diversity (H^1^) showed more or less higher value (>0.8) throughout the study period. Species richness was the highest (4.36) in March (Station 3). Diversity index showed higher value in Station 3 and 5 in the month of March. Species evenness (J) i.e., Species equitability recorded highest value (3.25) in Station 1 in the month of April. Species evenness was found to be higher (>1) throughout the study period (Figure 8).

## Discussion

### Chlorophyll-a as biomass index

Phytoplankton is a good indicator of trophic states; each and every change in environment affects this community. Many species of this community are very sensitive to changes and also they respond very quickly. We attempted to assess and predict the trophic state of the tidal creek on the basis of phytoplankton data. The phytoplankton biomass in water of Sundarban estuary was measured by chlorophyll-a concentration when typical seasonal growth pattern was observed in all stations. The phytoplankton biomass increased steadily from November 2008 to February 2009 and thereafter dropped sharply and ultimately reached at a minimum value in May 2009 (Figure 3). The phytoplankton cell density evidenced exactly similar pattern as phytoplankton biomass.

Similar community structure was also reported in Adriatic Sea [25] and in Boka Kotorska Bay [26] in Europe. Phytoplankton cycle reached its maximum in winter and slowed down gradually in summer season, which is perfectly similar to the universal pattern.

The factors that regulate biomass of planktons include nutrients like Nitrogen [27] Phosphorus [28] and Silica [29] but simultaneously high nutrient concentration also enhances the risk of Eutrophication.

**Figure 9:**
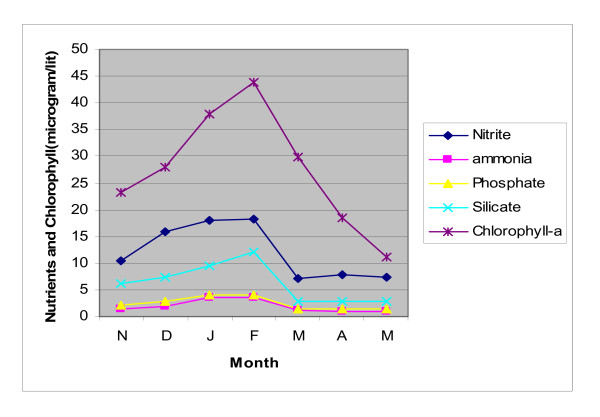
**Seasonal variation in chlorophyll-a concentration (μg L^-1^) with nutrients throughout the study period (Nov'08-May'09)**. Points represent mean of twenty samples in every month.

Dynamic relationship was noticed between level of these nutrients and Chlorophyll a concentration (Figure 9), correlation coefficients between these nutrient levels and chlorophyll a was determined. (Table 7). Coefficients with p-value < 0.2 had a significant relationship with chlorophyll-a [23]. Correlation coefficient determination yielded value of p much less than 0.2 for all the parameters and showed positive correlations signifying that these nutrients levels of nitrite + ammonia-nitrogen (N), phosphate (P) and silicate (Si) regulated phytoplankton biomass production in the estuary (Figure 9).

**Table 7:** Correlation coefficients

	N	P	Si	Tur	DO	Chl a
**N**	1					
**P**	0.9259	1				
**Si**	0.9533	0.9256	1			
**Tur**	-0.6007	-0.4556	-0.6512	1		
**DO**	0.6011	0.7240	0.6169	-0.1146	1	
**Chl a**	0.8252	**0.8356**	**0.8538**	**-0.6668**	**0.6102**	**1**

Correlation coefficients for various parameters such as nitrite + ammonia-nitrogen(N) Phosphorus(P), Silicate(Si), Dissolved oxygen(DO), Turbidity(Tur) with Chlorophyll-a.

A regression equation was explored to describe the interrelations amongst the parameters like nutrients, dissolved oxygen and turbidity and their combined effect on chlorophyll-a level. The pH and salinity level of water were not included in this equation as these variables showed negative correlation with chlorophyll level and p values were greater than 0.2. There were 150 observations in the data sets that include chlorophyll-a for possible use in the equation.

A sensitivity test was performed to identify the most dominant parameters. According to their importance in the equation the parameters were organized in descending order: phosphate, DO, turbidity, nitrite + ammonia-nitrogen, silicate.

[Chl a] = 10.34 + 5.31 [P] + 2.28 [DO]

-1.10 [Tur] - 0.18 [N] - 0.02 [Si]

R^2 ^= 0.81

Where, Chl a = concentration of Chlorophyll-a

DO = concentration of dissolved oxygen (mg L^-1^)

N = concentration of nitrite + ammonia-nitrogen (μg L^-1^)

P = concentration of total phosphorous (μg L^-1^)

Si = concentration of silicate (μg L^-1^)

Tur = turbidity (NTU)

**Figure 10:**
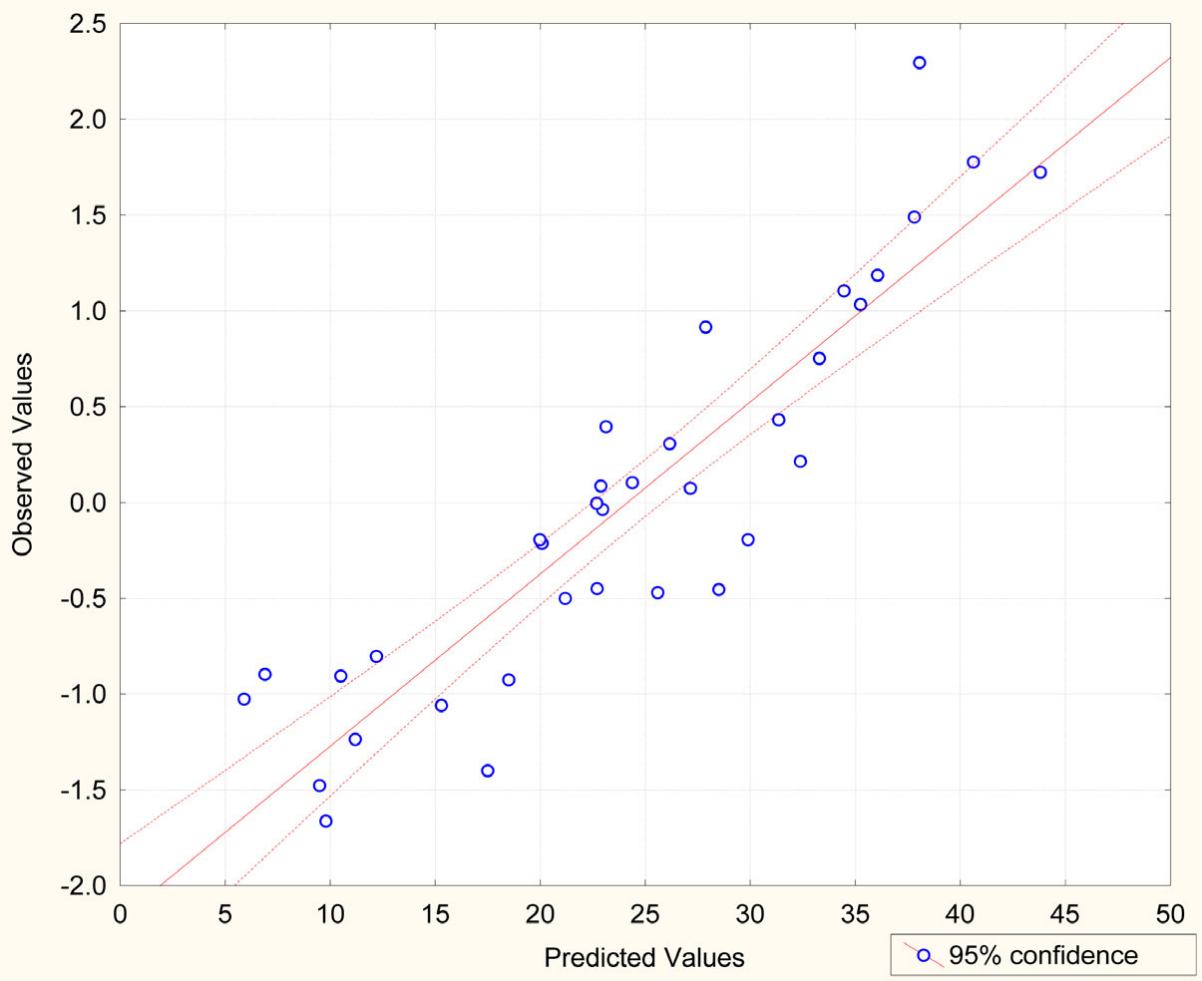
**Plot of observed versus predicted values of chlorophyll a from predicted equation**.

The coefficient of determination (R^2 ^= 0.81) was relatively high and the relationship described by this equation was highly significant (p < 0.0001). Actual versus predicted values for Chl *a *concentration using the data set in conjunction with the equation developed in this study provided a good prediction (Figure 10).

**Figure 11:**
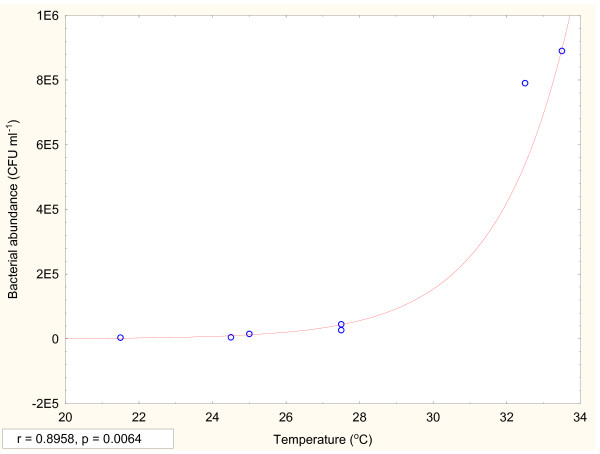
**Variation of bacterial abundance (CFU ml^-1^) with temperature (°C)**. Points represent mean of twenty samples in each month.

Biomass production gradually increased starting from November 2008 to February 2009 despite the fact that salinity of water also increased during the period; indicating that the salinity regime was within the tolerance level of planktonic biomass. During the month of March 2009 this pattern was discontinued and at salinity level 21.2 PSU the phytoplankton biomass declined in all stations indicating the tolerance level for majority of phytoplankton species. At this higher salinity level, a new set of euryhaline phytoplankton species (e.g. *Dunalielle salina, Trichodesmium, Chlorella salina *etc.) was observed which were resilient to higher salinity level [30-33]. The bacterial population (Table 3) was the lowest in January'09 (3.68 × 10^6 ^CFU L^-1^) and the highest in May'09 (8.9 × 10^8 ^CFU L^-1^) showing exponential relation with temperature (p = 0.006, r = 0.896) (Figure 11). The bacterial cell count obtained by fluorescence microscopy was higher than that of conventional method (plate count), as both culturable as well as nonculturable bacterial population were observed in fluorescence microscopy.

### Eutrophication of Estuary

The balance of water ecosystem is disturbed by eutrophication i.e. excessive fertilization, which, in turn, leads to increases in phytoplankton quantity and primary production. Eutrophication can have significant deleterious effects on the beneficial uses of estuarine and marine waters. Eutrophication also initiates changes in phytoplankton community structure, decrease in diversity and frequency of harmful algal blooms. Metrices based on phytoplankton quantity and productivity are widely used indicators of eutrophication in the status assessment of surface waters [34,35]. There are a number of ways in which eutrophication of estuary manifest itself: increase in phytoplankton biomass [36] and macroalgae [37] anoxia and hypoxia [38,39] even fish and benthos kill [40]. The most commonly used indicator of eutrophication in waterbody, however, is chlorophyll a [41].

During winter months (November 2008 - February 2009), the chlorophyll a concentration remained very high (>10 μg L^-1^) in all the five stations. This indicates that the estuary was in eutrophic condition during that time [42]. High nutrient input in coastal estuary water may be responsible for successful establishment and enhancement of nuisance algal species like Cyanophyceae and Dinophyceae [43]. The chlorophyll a level dropped rapidly at the onset of summer and reached a comparatively lower value (8.66 μg L^-1^) in May. Thus the estuary was mesotrophic-eutrophic (<10 μg L^-1^) in summer.

The poor water quality of the estuary could be ascertained from the presence of toxic Dinoflagellates like *Dinophysis *[44], *Polykrikos *[45], *Prorocentrum *[46], Cyanophyceae like *Anabaena, Oscillatoria *[47] and diatom like *Nitzschia *[47]. Many studies on mangrove sediments of Sundarban also indicated the occurrence of pollutant chemicals in Sundarban region [48-51].

Eutrophication seems to be a global problem. Nutrient-enrichment of the coastal zone increases the mortality of mangroves by enhancing shoot growth relative to root which makes them vulnerable to environmental stresses like salinity, drought that adversely affect plant water relationships [52]. The Eutrophication of this tidal creek may have detrimental effect on the mangrove vegetation.

### Community Structure and biodiversity

The Sundarbans estuarine phytoplankton community is rich in species diversity and species richness [53]. In all the five stations, throughout the study period, species diversity and species richness showed a value >0.8 indicating healthy phytoplankton community in the estuary. However, the diversity index increased steadily from November 2008 to March 2009 and ultimately reached its peak (2.5) in March and then declined slightly. The species richness index also followed the same pattern but attained the maximum in the month of April, 2009.

A high value of diversity index generally implies healthy ecosystem while a low value indicates degraded state. Investigation was made to find out a relationship between the trophic state of ecosystem and species diversity. It was observed that during post monsoon (November 2008 - February 2009) when species diversity increased steadily, the phytoplankton biomass (chlorophyll-a > 10 μg L^-1^) also increased in all the stations and the estuary was in a eutrophic state [54]. During premonsoon (March-May, 2009), the species diversity index declined and so also the phytoplankton biomass.

However, phytoplankton diversity depends on a number of factors other than nutrient supply. For instance, at a higher trophic level, the impact of predation of fish constitutes a strong top down control on phytoplankton assemblage [55]. The pollution status of water also strongly influences the phytoplankton species diversity. Thus, phytoplankton diversity index should not be accounted as a good indicator of trophic state of an estuary. The diversity indices very well characterize the differences between assemblages and associations, though the factors which influence diversity are seldom governed by trophic state [23].

## Conclusion

The dyamics of Sundarban is mainly maintained by sedimentations from the three major rivers Ganga, Bramhaputra and Meghna. Both the spatial and temporal influences have been demonstrated on the salinity in this region. While describing mangrove forest of Sundarbans, general tendency is to describe this forest as 'Pristine' and totally devoid of any human intervention [56]. However, detail regional study using remote sensing technique on a temporal scale coupled with extensive ground truth survey points to the fact that in the fringe areas of these forested islands with inhabited islands, human intervention is not very rare; rather in some cases, the change in land cover condition is so pronounced that it may be termed as to cross the threshold limit. The most glaring examples of such human interventions have been identified in the Herobhanga Forest Block along the creek. That is precisely the reason behind identification of this area as study area so as to estimate the effect of human intervention on the overall mangrove eco-system of Sundarbans.

The estuary remained eutrophic for most time of the year and mesotrophic-eutrophic during the summer months. Overload of nutrients flowing into the estuary resulted in high levels of dissolved Nitrogen, Phosphorus and Silicate and supporting high algal growth. The Correlation coefficient (p ≪ 0.2) for all the parameters signify that these components play crucial role to drive the trophic level of the estuary. The coefficients of the regression equation indicate that phosphate, DO, turbidity, nitrogen and silicate control the production of algal biomass (and hence eutrophic state) in decreasing order of influence.

The trend of biomass production of phytoplanktons represents that specific threshold was reached at salinity level around 21 PSU. Not only the biomass production decreased beyond this salinity level, the species assemblage also changed in favour of more salinity tolerant species. Although the bacterioplankton count did not exhibit similar profile. Phytoplankton abundance was in the range of 1.80 × 10^4 ^cells L^-1 ^- 2.05 × 10^7 ^cells L^-1 ^and bacterial population was in the range of 8.54 × 10^7^cells L^-1^- 4.52 × 10^10 ^cells L^-1 ^in the estuary throughout the study period. Phytoplankton community was observed to be dominated by diatoms (Bacillariophyceae) followed by Pyrrophyceae (Dinoflagellates) and Chlorophyceae A total of 46 taxa belonging to six algal groups were identified from the estuarine water of Sundarban.

The high species diversity and species richness of phytoplanktons in the estuary throughout the study period indicated overall good health of the producers in the ecosystem. However, during pre-monsoon period, the species diversity and species richness showed declining trend that possibly resulted from stress in the abiotic environment.

Mangroves are the only woody halophytes dominated ecosystem situated at the confluence of land and sea, they occupy a harsh environment, being daily subject to tidal changes in temperature, water and salt-exposure and varying degree of anoxia [57]. Eutrophication as well as presence of toxic Dinoflagellates and Cyanophyceae in the tidal creek of Sundarban estuary definitely revealed the deteriorated status of the water quality.

Mangrove communities are recognized as highly productive ecosystems that provide large quantities of organic matter to adjacent coastal waters in the form of detritus and live animals [58]. There is a close microbe-nutrient-plant relationship that functions as a mechanism to recycle and conserve nutrients in the mangrove ecosystem. Nutrient enrichment is one of the most serious threats to near shore coastal ecosystems. Increasing nutrients availability introduces an instability into mangrove forests that lowers their resilience to environmental variability. The instability arises because nutrients, particularly nitrogen, stimulate growth of shoots relative to roots [59], thereby enhancing productivity during favorable periods but increasing vulnerability to water stress during drought. Enhanced instability with coastal eutrophication has far reaching consequences for many aspects of mangrove ecosystem function under contemporary and future climate conditions [52]. Apart from other aspects this study gains its significance for throwing light on future of one of the most precious natural resource of this biogeographic region.

## Competing interests

The authors declare that they have no competing interests.

## Authors' contributions

SM and KC performed all experiments, calculated results, prepared the tables, graphs and diagrams, and composed the draft manuscript in consultation with MB. MB designed the experiments, analyzed and interpreted data and results, modified the manuscript in the final form. SB planned the project, was involved in acquisition of funds, selected the site and field stations according to GPS measurement, prepared the map, supplied remote sensing information, and guided the field study and sample collection. All the authors read and approved the final manuscript.
